# Regulation of Nutrient Transport across the Placenta

**DOI:** 10.1155/2012/179827

**Published:** 2012-12-10

**Authors:** Susanne Lager, Theresa L. Powell

**Affiliations:** Department of Obstetrics and Gynecology, Center for Pregnancy and Newborn Research, University of Texas Health Science Center at San Antonio, 7703 Floyd Curl Drive, Mail Code 7836, San Antonio, TX 78229-3900, USA

## Abstract

Abnormal fetal growth, both growth restriction and overgrowth, is associated with perinatal complications and an increased risk of metabolic and cardiovascular disease later in life. Fetal growth is dependent on nutrient availability, which in turn is related to the capacity of the placenta to transport these nutrients. The activity of a range of nutrient transporters has been reported to be decreased in placentas of growth restricted fetuses, whereas at least some studies indicate that placental nutrient transport is upregulated in fetal overgrowth. These findings suggest that changes in placental nutrient transport may directly contribute to the development of abnormal fetal growth. Detailed information on the mechanisms by which placental nutrient transporters are regulated will therefore help us to better understand how important pregnancy complications develop and may provide a foundation for designing novel intervention strategies. In this paper we will focus on recent studies of regulatory mechanisms that modulate placental transport of amino acids, fatty acids, and glucose.

## 1. Introduction 

Altered fetal growth represents an important risk factor for complications in the perinatal period [1, 2] and is associated with the development of cardiovascular disease, obesity, and diabetes in adult life [3–7]. Fetal growth is largely determined by nutrient supply, which is dependent upon placental nutrient transport. In human pregnancies complicated by either intrauterine growth restriction (IUGR) or fetal overgrowth many key placental nutrient transporters are specifically regulated (Table 1). This suggests that changes in placental nutrient transport directly contribute to altered fetal growth. Information on molecular mechanisms regulating placental nutrient transporters is critical for understanding the development of pregnancy complications, as well as how maternal nutrition and metabolic disturbances affect fetal growth. Furthermore, the placenta with its nutrient transport functions is increasingly seen as being the mediator of maternal nutrition effects on the lifelong health consequences for the child [8–10].

Several factors influence transport across the placenta: uteroplacental and umbilical blood flows, area available for exchange, placental metabolism, and activity/expression of specific transporter proteins in the placental barrier. Transfer of highly permeable molecules such as oxygen and carbon dioxide is particularly influenced by reduced blood flow [11]. For less permeable substrates the placenta possesses both passive and active transport mechanisms. Glucose crosses the placenta by facilitated diffusion. Net glucose transfer is therefore highly dependent on maternal-fetal concentration gradients [12]. Other nutrients (e.g., calcium) are transported by primary active transport; a process directly linked to the hydrolysis of adenosine triphosphate [13]. A wide range of nutrients (e.g., amino acids, phosphorus, and lactate) are transported across the placenta mediated by secondary active transport, utilizing energy provided by ion gradients such as sodium, chloride, and protons [14–16]. Changes in energy availability or ion gradients can profoundly influence net transfer of substrates transported by active mechanisms. In this paper, we will focus on circulating and placental factors as well as placental signaling pathways regulating key placental macronutrient transporters. 

## 2. Factors Regulating Placental Nutrient ****Transporters: An Overview 

**Figure 1:**
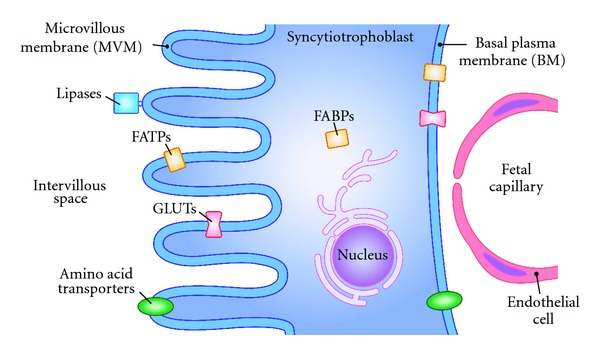
The syncytiotrophoblast represents the primary barrier for transfer of nutrients from mother to fetus. Maternal blood pools in the intervillous space and bathes the microvillous membrane (MVM). The basal plasma membrane (BM) of the syncytiotrophoblast is oriented toward the fetal circulation. Transporters mediating the transfer of amino acids, glucose (GLUTs), and fatty acids (FATPs) are expressed in both plasma membranes of the syncytiotrophoblast. For transfer of lipids, extracellular lipases release fatty acids from maternal lipoproteins and intracellular binding proteins (FABPs) guide the fatty acids within the cytosol of the syncytiotrophoblast.

**Figure 2:**
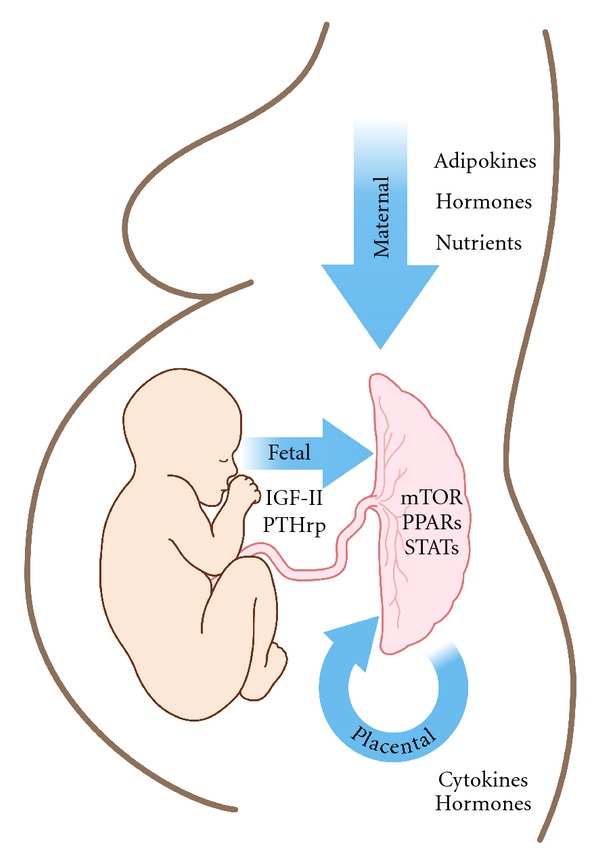
Placental function is likely regulated by numerous factors of fetal, maternal, and placental origin. Fetal signals affecting placental function include IGF-II and PTHrp. The effects of maternal factors, including adipokines, hormones, and nutrient levels, on placental function have been investigated in various models such as cultured trophoblast cells, placental explants, and perfused placenta. The placenta also expresses a wide array of molecules which are released into both fetal and maternal circulations. These substances may impact on the placenta itself in an autocrine/paracrine fashion. Integration of numerous, and sometimes divergent, signaling by intracellular regulatory pathways such as mTOR, PPAR, and STAT has been demonstrated to affect placental nutrient transport.

The primary barrier limiting nutrient transfer across the human placenta is the syncytiotrophoblast [9, 17]. The syncytiotrophoblast has two polarized plasma membranes: a maternal-facing microvillous plasma membrane (MVM) and a basal plasma membrane (BM) oriented towards the fetal circulation (Figure 1). Transport characteristics of these two plasma membranes will have a major influence on net placental nutrient transport and consequently fetal growth. The syncytiotrophoblast plasma membranes express numerous nutrient transporters which may be regulated by fetal, maternal, and placental signals (Figure 2).

Significant support for fetal demand signals regulating placental amino acid transport comes from studies of mice with placenta specific knockout of the insulin-like growth factor 2 gene (*Igf2*). Placental growth restriction occurs in midgestation and system A amino acid transporter activity is initially upregulated in this model. The increased nutrient transport maintains fetal growth within the normal range until late pregnancy when compensatory mechanisms fail and IUGR develops [18, 19]. At least some studies in humans have shown that IGF-II levels are reduced in IUGR fetuses [20] and higher in large for gestational age (LGA) fetuses [21]. The finding that MVM system A amino acid transporter activity is inversely correlated to fetal size within the normal range of birth weights provides indirect evidence for fetal regulation of placental transport functions [22]. In human pregnancy, studies suggest that fetal parathyroid hormone-related peptide (PTHrp) regulates activity of the calcium pump in the BM [23, 24]. 

Placental signals originating from imprinted genes have been shown to regulate nutrient transport in the mouse placenta. Imprinted genes are predominantly expressed from one of two parental alleles, and in mice approximately 70 imprinted genes have been discovered [25]. A subgroup of these genes are imprinted only in the placenta and are involved in regulation of fetal and placental growth [25]. An example of a paternally expressed/maternally repressed placental gene is *Igf2 *[19]. * *IGF-II regulates placental growth and therefore indirectly its transport capacity. Interestingly, system A or sodium-dependent neutral amino acid transporter (SNAT) isoform 4 (encoded by *Slc38a4*) is expressed from the paternal allele in mouse placenta [25, 26]. Thus, the paternal genome may exert influence on placental amino acid transport capacity and fetal growth by means of paternally imprinted placental genes. In addition to imprinted genes, numerous placental signaling pathways, such as mammalian target of rapamycin (mTOR), regulate nutrient transporters. Furthermore a wide array of cytokines, hormones, and signaling molecules are synthesized and secreted by the placenta and could affect its nutrient transporters in an autocrine/paracrine fashion. 

There is strong support for regulation of placental nutrient transport by maternal factors, particularly by metabolic hormones. The MVM of the syncytiotrophoblast expresses numerous hormone receptors, including receptors for insulin [27, 28], IGF-I [29], and leptin [30], consistent with regulation of trophoblast function by maternal hormones. In subsequent sections, we will review circulating factors, placental factors, and placental signaling pathways known to regulate placental transporters for amino acids, fatty acids, and glucose. We will also discuss changes in activity and expression of placental nutrient transporters in pregnancies complicated by abnormal fetal growth. The primary focus will be on data obtained from studies of human placenta; animal studies will be discussed as appropriate, primarily when human data is lacking. 

## 3. Amino Acids 

Amino acid uptake across cellular membranes is mediated through active transporter processes; these mechanisms can be separated into accumulative transporters and exchangers [31]. The accumulative transporters increase intracellular amino acid concentrations by mediating uptake against their concentration gradient, usually by cotransporting extracellular sodium [31]. Exchangers alter the intracellular amino acid composition by exchanging amino acids between the intracellular and extracellular compartments [31, 32]. Amino acid transporters can be further categorized into distinct systems depending on characteristics such as substrate specificity and sodium dependence [31]. The human placenta expresses more than 20 different amino acid transporters [14, 33]. Fetal amino acid concentrations are generally higher than maternal levels [34], reflecting an active transport mechanism across the placenta. Efflux of amino acids across the BM has been suggested to be dependent on amino acid exchangers together with facilitative efflux transporters [33]. 

### 3.1. System A

The system A amino acid transporter facilitates uptake of small non-essential neutral amino acids such as alanine, glycine, and serine [14]. This transporter system enables uptake of amino acids against their concentration gradient by simultaneously transporting sodium into the cell. System A activity has been measured in both plasma membranes of the syncytiotrophoblast, however, highly polarized to the MVM [35]. Importantly, system A activity contributes to the high intracellular concentration of amino acids such as glycine, which are exchanged for extracellular essential amino acids via system L (see the following). System A activity is thus important for placental transport of both non-essential and essential amino acids.

Human first and third trimester placenta express three isoforms of system A: SNAT1 (*SLC38A1*), SNAT2 (*SLC38A2*), and SNAT4 (*SLC38A4*) [36]. During pregnancy the relative contributions of the three isoforms to total system A activity may vary. Using isolated MVM and villous fragments, Desforges et al. have provided evidence suggesting that the SNAT4 contribution is higher in first trimester compared to term placenta [36]. Additionally, SNAT1 is the major contributor to system A activity in cultured primary trophoblast cells from term placentas [37]. System A activity has been reported to increase with advancing gestation [36, 38], but conflicting results have been reported [39]. 

Placental system A transporter activity is reduced in human pregnancies complicated by IUGR [35, 40]. However, it is unclear whether reduced amino acid transport occurs prior to fetal growth restriction or as a consequence of decreased fetal needs. In animal studies of growth restriction, reduction in system A activity has been shown to precede impaired fetal growth [41, 42]. These studies suggest alterations in system A transporters may be contributing to the development of IUGR.

#### 3.1.1. Regulation of Placental System A Activity

Factors such as cytokines and hormones regulate placental system A activity* in vitro*. Insulin [43–46], interleukin (IL)-6 [47], and tumor necrosis factor (TNF)-*α* [47] stimulate activity of this transporter. Importantly, the stimulatory effect of insulin, IL-6, and TNF-*α* occurs at concentrations within the physiological range. The mechanism through which TNF-*α* stimulates system A is unknown, but the effect of IL-6 is dependent on signal transducer and activator of transcription (STAT)3 [47]. The involvement of STAT3 in regulating placental amino acid transport has also been demonstrated in villous fragments where STAT3 is required for stimulation of system A activity by leptin [45]. Preliminary studies show oleic acid activates STAT3; this fatty acid also increases system A activity through a toll-like receptor-4 dependent mechanism in cultured trophoblast cells [48]. 

Another placental signaling pathway regulating placental system A activity is mTOR, which we have suggested functions as an integrator of maternal, fetal, and placental signaling molecules (e.g., hormones, growth factors, and nutrient levels) [49]. mTOR is an important regulator of growth for individual cells as well as whole organs. In placenta from pregnancies complicated by IUGR, activity of mTOR signaling is reduced [50, 51]. Obese women have an increased risk of delivering large babies [52], and placental mTOR activity increases in such pregnancies [53]. In cultured trophoblast cells, mTOR functions as a positive regulator of system A activity [46, 54] without affecting global expression of the three SNAT isoforms [54], but rather at the posttranslational level by affecting plasma membrane trafficking of the SNAT2 isoform [55]. 

*In utero* exposure to glucocorticoids is associated with reduced fetal growth [56], and glucocorticoids affect placental system A activity. Dexamethasone (a synthetic glucocorticoid) stimulates system A activity in cultured primary trophoblast cells [57] and in explants from term placenta [58]. Cortisol also increases system A activity in the BeWo trophoblast cell model [59]. This stimulatory effect may depend upon cortisol's ability to increase SNAT2 expression [59] because a 1-hour exposure does not alter system A activity in placental villous explants [43]. Notably, dexamethasone administered to pregnant mice downregulates placental system A amino acid transport [60] suggesting the effects of glucocorticoids on system A activity *in vitro* and *in vivo* are distinct. 

Leptin stimulates placental system A activity [43, 45], yet the effect appears limited to acute stimulation because longer exposures did not alter amino acid uptake [45, 61]. Contrarily, the stimulatory effect of insulin is more robust, since insulin stimulates system A activity after both acute and longer exposures [43–46]. Insulin's stimulatory effect on system A activity is inhibited by full-length adiponectin [44]. In contrast to other tissues, this implies adiponectin causes placental insulin resistance rather than promoting increased insulin sensitivity. The form of adiponectin is also important since globular adiponectin increases system A activity, while full-length adiponectin alone does not [44]. Interestingly, infusing pregnant mice with full-length adiponectin results in reduced placental system A activity and restricted fetal growth [62]. 

 Although it has been suggested that the insulin receptor is expressed primarily in endothelial cells of fetal capillaries at term [96], recent evidence suggests otherwise. The insulin receptor is in fact highly expressed in the MVM at term [97]. This finding suggests maternal insulin could affect trophoblast function *in vivo*. IGF-I stimulates system A activity and can initiate signaling through both IGF-I and insulin receptors [46, 78]. In a two-sided model, Bloxam et al. demonstrated trophoblast cells exposed to IGF-I on the apical side increased intracellular accumulation of AIB (a system A substrate) but also reduced overall transfer rate [98]. Although MVM uptake is the first step in transferring nutrients across the placenta, this finding highlights that increased uptake may not always result in an increased net transport. 

Other factors are reported to reduce placental system A activity, including hypoxia [99] and IL-1*β* [100]. In explants from term placenta, acute but not chronic exposure to angiotensin II reduced activity of the system A transporter [61]. Blocking the sodium pump with ouabain or subjecting villous explants to a chronic exposure to urocortin and corticotropin-releasing hormone (CRH) also cause reduction in system A activity [61]. 

Mechanisms regulating amino acid transport may very well differ in early versus late pregnancy. While most studies are performed with term placenta tissue, first or second trimester placenta may respond differently to stimuli. For example, growth hormone (GH) reduced system A activity in villous tissue from first trimester [39], but not those from term placenta [43]. Conversely, insulin has no effect in early pregnancy while it stimulates activity of system A transporter in third trimester [39, 43]. 

### 3.2. System L

The system L transporter is a sodium-independent obligatory exchanger of neutral amino acids. Non-essential amino acids are exchanged for predominantly essential amino acids, with either aromatic or branched side chains (e.g., leucine and phenylalanine) enabling transport against their concentration gradient [32]. The system L transporter consists of a heterodimer formed from a light chain protein: large neutral amino acid transporter (LAT)1 (*SLC7A5*) or LAT2 (*SLC7A8*) together with a heavy chain transmembrane protein: 4F2hc/CD98 (*SLC3A2*) [32, 101]. Expression of 4F2hc and LAT1 is higher in term placenta compared to midgestation placenta [101]. Cellular localization of the LAT isoforms is polarized, with LAT1 primarily found in the MVM and LAT2 in the BM [101–103]. Although BM may be the primary localization of LAT2, it is not exclusively expressed in this plasma membrane since functional activity of LAT2 has been demonstrated in isolated MVM vesicles [104]. The BM localization of LAT2 allows for exchange of amino acids between non-essential amino acids in the fetal compartment with essential amino acids in the cytoplasm. The BM also expresses the efflux transporters TAT1 (*SLC16A10*), LAT3 (*SLC43A1*), and LAT4 (*SLC43A2*); these transporters may play an important role in the net efflux of amino acids to the fetus [105]. Similar to the system A transporter, activity of the system L transporter has been reported in both plasma membranes of the syncytiotrophoblast, although with higher activity measured in the MVM [103]. The activity of system L in the MVM decreases in cases of reduced fetal growth, both in human [63] and animal models [42, 62]. 

#### 3.2.1. Regulation of Placental System L Activity

In cultured primary trophoblast cells system L amino acid transporter activity is not affected by factors known to regulate system A activity (e.g., IL-6 [47], TNF-*α* [47], or adiponectin [44]). Yet, in a pregnant mouse model, a four-day infusion of full-length adiponectin caused reduced fetal growth and downregulation of system L activity as well as expression of the two LAT isoforms [62]. Conflicting results have been reported for insulin regulation of system L activity. In one study, insulin modestly increased system L activity in cultured primary trophoblast cells [46], whereas no effect was observed in another study using similar approaches [44]. 

Studies of cultured trophoblast cells [54] and villous explants [50] from term placenta have demonstrated that the mTOR signaling pathway functions as a positive regulator of placental system L activity. Exposing trophoblast cells to rapamycin, an mTOR inhibitor, reduces system L activity [50, 54] and decreases LAT1 mRNA expression [54]. Furthermore, mTOR inhibition prevented the increase in leucine uptake observed after incubation of primary human trophoblast cells in insulin or glucose [46]. 

Activation of protein kinase C together with increased intracellular calcium levels in BeWo cells increases expression of 4F2hc and LAT1 and stimulates system L activity [101]. Cocaine and nicotine use during pregnancy are associated with lower birth weights, resulting in studies exploring the effects of such drugs upon placental amino acid transport. With human placenta perfused *in vitro*, cocaine (or a combination of cocaine and nicotine) reduced maternal-fetal transport of amino acids mediated by system L, system A, and system y^+^ transporters [106].

### 3.3. Taurine

Taurine is regarded as essential for the fetus because the ability to synthesize this amino acid is low or absent during fetal life. The taurine transporter (system *β*, TauT; *SLC6A6*) mediates cellular taurine uptake against its concentration gradient energized by cotransport with sodium and chloride. The taurine transporter is more active in the MVM compared to the BM [64]. Activity of the taurine transporter has been reported as reduced in MVM, but not BM, isolated from IUGR placentas [64]. 

#### 3.3.1. Regulation of Placental Taurine Transport

In pregnancies complicated by IUGR, it is believed that the placenta is subjected to oxidative stress [92] and fetal nitric oxide levels (measured in venous umbilical cord blood) are elevated [107]. The nitric oxide donor SIN-1 has been shown to reduce taurine transport in both isolated MVM vesicles and villous tissue explants [65, 93]. Uptake of taurine in villous tissue from human placenta is not affected by cytokines or hormones, such as IGF-I and II, IL-1*β*, IL-6, GH, leptin, or TNF-*α* [65]. Furthermore, taurine uptake in villous tissue explants was not affected by inhibiting mTOR [65]. In contrast, mTOR has been shown to be a positive regulator of taurine transport in cultured trophoblast cells; it also affects mRNA expression of this transporter [46, 54]. High glucose levels decrease taurine uptake in cultured trophoblast cells. The effect may be independent of mTOR because glucose deprivation stimulates taurine uptake both in the absence and presence of rapamycin [46]. 

## 4. Fatty Acids 

Fatty acids are a source of energy, constitute an essential structural element of cellular membranes, and are precursors for important bioactive compounds. Fatty acids are important for development of specific tissues and organs. For instance, in the third trimester, fat rapidly accumulates in fetal adipose tissue and brain [17]. The long-chained polyunsaturated fatty acids (LCPUFAs) are of particular importance for brain development [108, 109]. Fatty acids taken up by the placenta and transported to the fetus originate predominantly from two sources in maternal circulation: nonesterified fatty acids (NEFAs) and esterified fatty acids in triglycerides (TGs) carried by lipoproteins. Maternal TGs have been suggested as a primary source of fatty acids because of their substantial increase in late gestation compared to NEFAs [17]. 

### 4.1. Placental Lipases

Fatty acids (in the form of TGs) transported in maternal lipoproteins are made available for placental transport by hydrolysis into NEFAs. This enzymatic step is accomplished by lipases associated with the MVM. The placenta expresses several lipases [110], two of which function in the extracellular compartment: lipoprotein lipase (LPL; *LPL*) and endothelial lipase (EL; *LIPG*) which is consistent with reports of two different lipase activities identified in isolated MVM vesicles [111]. LPL activity and/or expression in cultured trophoblast cells, MVM vesicles, or placental villous tissue have been reported by several groups [66, 67, 79, 81, 89, 110–112]. However, conflicting data on localization of LPL to the syncytiotrophoblast in term placenta exists [68]. 

LPL activity per gram tissue in villous explants from term placenta is higher compared to first trimester [79], suggesting placental LPL activity increases with advancing gestation. Pregnancy complications also affect placental LPL. The mRNA expression of LPL is increased [67], but measurable placental LPL activity is reduced in IUGR babies born preterm [66]. LPL activity is increased in MVM isolated from placenta of pregnancies complicated by maternal insulin-dependent diabetes with fetal overgrowth [66]. 

LPL activity and expression is regulated in a tissue-specific manner [113]. Placental LPL activity is reduced by chronic exposure to cortisol, estradiol, IGF-I, or insulin [79]. Shorter exposures to physiological concentrations of estradiol and insulin in combination with hyperglycemia increase LPL activity [79]. In cultured trophoblast cells, LPL activity is reduced by TGs and NEFAs [89]. The mechanism for the reduced activity is unknown, but it may occur through displacement of the LPL enzyme from its extracellular anchoring site [89]. Cytokines have been shown to regulate LPL activity in other tissues [113]; however, TNF-*α* does not modulate LPL activity in villous explants [79] or in cultured trophoblast cells [81]. In addition, maternal late pregnancy IL-6 levels are inversely correlated with placental LPL activity at term. However, in cultured trophoblast cell experiments, IL-6 does not directly regulate activity of this lipase [81]. Collectively, these data suggest that placental LPL is not regulated by proinflammatory cytokines. 

While LPL primarily hydrolyses TGs in chylomicrons and very low-density lipoprotein particles, high-density lipoprotein phospholipids are the preferred substrate of EL. In placenta, both endothelial cells and the syncytiotrophoblast have been reported to express EL [112]. The EL expression changes during pregnancy; term placenta expresses higher levels of EL than first trimester placenta [68], and pregnancy complications have also been shown to influence placental EL expression. Maternal obesity combined with gestational diabetes results in higher placental expression of EL [114]; contrastingly, placentas of IUGR pregnancies have decreased EL expression [68]. 

### 4.2. Transporters of Fatty Acids

Transfer of fatty acids from mother to fetus may be driven by the maternal to fetal concentration gradient [17]. NEFAs may cross lipid bilayers by simple diffusion, but to what degree cells rely on this diffusion for fatty acid uptake is unclear [115–117]. In tissues with high fatty acid demand, uptake of fatty acids by simple diffusion may be insufficient to meet minimum requirements [118]. To what extent NEFAs diffuse across the syncytiotrophoblast's plasma membranes and contribute to fatty acid transfer to the fetus is currently unknown. Preferential accumulation (biomagnification) of LCPUFAs in the fetal compartment [119, 120] is consistent with a mediated transport of these fatty acids. There is evidence for selective transport of LCPUFAs in cell culture models [121, 122] and perfused placenta [123], as well as from *in vivo* studies with docosahexaenoic acid (DHA) labeled with stable isotopes [124]. 

Fatty acid transport proteins (FATPs) are integral membrane proteins important for cellular uptake of long-chained fatty acids [118]. The FATP family consists of six related members, of which five are expressed in human placenta (FATP1-4, and 6; *SLC27A1-4, *and* 6*) [125]. At the mRNA level, FATP2, FATP4, and FATP6 are more highly expressed [126]. FATP1 protein expression has been demonstrated in both BM and MVM [127, 128], but cellular localization of the other isoforms in placenta is currently unknown. Placental FATP1 and FATP4 mRNA expression correlates with DHA levels in both maternal plasma and placental phospholipids [129]. Only FATP4 mRNA expression correlated with fetal DHA phospholipid levels, suggesting FATP4 is particularly important for placental LCPUFA transfer [129]. Studies exploring mechanisms regulating placental FATPs have been focused primarily on mRNA expression levels. In cultured trophoblast cells from term placenta, peroxisome proliferator-activated receptor (PPAR)*γ*/retinoid X receptor signaling and hypoxia regulate FATP1, FATP2, and FATP4 mRNA expression [125, 126]. In the same model, FATP4 mRNA expression was shown to be downregulated by chronic exposure to IL-6, but without corresponding alteration in protein expression [81]. It has been reported in animal models of maternal obesity that placental mRNA expression of FATP1 and FATP4 are increased [130, 131].

In addition to FATPs, there are two other membrane-associated proteins expressed in placenta with the ability to transport fatty acids: a placenta specific membrane bound fatty acid binding protein (pFABPpm) and fatty acid translocase (FAT; *CD36*) [127]. pFABPpm is exclusively expressed in the MVM [127]. Functionally, pFABPpm exhibits a high affinity for LCPUFAs, suggesting this transporter is involved in preferential uptake of these fatty acids for transfer across the placenta [121]. Since proportionally less DHA and arachidonic acid is transferred to the fetus in IUGR pregnancies [132], but expression of FAT is unchanged [133], a role for FAT in selective LCPUFA transfer seems unlikely [134]. Little is known about regulation of pFABPpm and FAT in human placenta, but in cultured trophoblast cells, elevated levels of leptin cause an increase in FAT expression [87]. 

Fatty acid binding proteins (FABPs) within the cytosol of the syncytiotrophoblast traffic fatty acids to sites within the cell for esterification, beta-oxidation, or transfer to the fetus. Human placenta expresses four different isoforms of FABPs: FABP1, FABP3, FABP4, and FABP5 (*FABP1, 3, 4,* and *5*) [135]. In placentas from pregnancies complicated by maternal diabetes, FABP1 protein expression is increased [66]. This augmented FABP1 expression together with higher LPL activity has been suggested to contribute to increased fetal fat accumulation in pregnancies complicated by maternal diabetes [66]. In primary human trophoblast cells, FABP1 expression was upregulated by hypoxia and through PPAR*γ* signaling [135]. Hypoxia and/or PPAR*γ* signaling also increases expression of FABP3 and FABP4 [135]. Exposure to elevated levels of fatty acids increases FABP4 expression [90]. While FABP3 expression is not altered in pregnancies complicated by IUGR or maternal diabetes [66], FABP4 expression is increased in placenta from obese diabetic women [90]. 

## 5. Glucose 

Glucose is the major energy substrate for both fetus and placenta. Fetal glucose production is minimal; therefore, it is entirely dependent upon placental supply of glucose from the maternal circulation. Placental transport of glucose is mediated by facilitated carrier-mediated diffusion through specific glucose transporter proteins (GLUTs). GLUTs are expressed in both plasma membranes of the syncytiotrophoblast, with glucose transport occurring only down its concentration gradient. Higher maternal glucose concentrations compared to fetal drive net glucose transport toward the fetus. A high density of GLUTs in the MVM, together with the large surface area, allow for rapid glucose uptake into the syncytiotrophoblast. The high capacity of the MVM for glucose transport provides adequate glucose for placental consumption (corresponding to approximately one-third of total placental glucose uptake) while maintaining a gradient between the syncytiotrophoblast cell interior and the fetal capillary, which is necessary for net transport to the fetus. The GLUTs are expressed at a much lower level in the BM than the MVM. The lower BM GLUT expression, along with the surface area difference, has led to the proposal that BM transport constitutes the rate limiting step in placental glucose transfer [12]. 

GLUT1 *(SLC2A1)* is believed to be a primary glucose transporter isoform in the human placenta at term, and its expression increases over gestation [136]. GLUT1 has been localized to both plasma membranes, but with three-fold higher expression in the MVM [137]. Expression of GLUT1 in BM has been reported to increase in maternal diabetes [72, 138], resulting in increased BM glucose transport activity with this pregnancy complication [72]. GLUT1 is not the only glucose transporter expressed in human placenta. Despite previous reports of little or no GLUT3 *(SLC2A3)* expression in the syncytiotrophoblast of term placentas [136, 137, 139], GLUT3 expression has recently been reported throughout gestation in the syncytiotrophoblast, albeit with the highest expression in early pregnancy [140]. The human placenta also expresses GLUT8 *(SLC2A8) *[141], GLUT9 *(SLC2A9)* [142], and GLUT10 *(SLC2A10)* [143]. Interestingly, first trimester syncytiotrophoblast also expresses the insulin sensitive glucose transporter isoforms GLUT4 *(SLC2A4)* and GLUT12 *(SLC2A12) *[136, 144]. 

### 5.1. Regulation of Glucose Transport

Placental glucose uptake increases with advancing gestation [39], and regulation of glucose transport differs between trimesters. For instance, GH stimulates glucose uptake in villous tissue fragments from term placenta. However, GH has no effect on glucose uptake in first trimester villous fragments [39]. In cultured trophoblast cells from term placenta, CRH has opposing effects on GLUT1 and GLUT3 expression (increased GLUT1 and decreased GLUT3) [73]. CRH can act through two different receptors: CRH-R1 and CRH-R2. CRH-R1 expression decreases in IUGR placenta [145], and in cultured trophoblast cells, CRH-R1 positively regulates GLUT1 expression [73]. Because CRH is produced locally in the placenta [74], such data is consistent with autocrine/paracrine regulation of placental glucose transport mediated by CRH. 

Steroid hormones can also affect activity/expression of placental glucose transporters in term placenta. In studies measuring glucose uptake in isolated MVM vesicles, estrogens and progesterone were found to have a negative impact on glucose uptake rates [146]. In cultured trophoblast cells, glucocorticoids reduce the expression of GLUT1 and GLUT3 [147]. 

The effect of insulin on regulating placental glucose transport is gestational age dependent, and there are conflicting reports on its effects at term. Insulin does not affect glucose uptake in perfused term placenta [148] or in term villous fragments [39, 136]. However, other investigators have reported stimulation of placental glucose uptake by insulin at term [83, 84]. Ericsson and coworkers found that insulin stimulates glucose uptake in villous fragments from first trimester placenta but not from term placenta [136]. This observation could be due to the finding that GLUT4 and 12, two insulin-sensitive isoforms, are expressed in first trimester [136, 144] but not term placenta. Glucose levels also affect uptake by altering GLUT expression. Exposing cultured trophoblast cells to hyperglycemia reduced uptake rates and GLUT1 expression [76]. Depriving the cells of glucose has the opposite effect, causing increased uptake and expression of GLUT1 [76]. 

Oxidative stress not only affects placental transport of amino acids [65, 93], but can also influence transport of glucose. In placental explants, increased oxidative stress resulted in reduced glucose uptake and lowered GLUT1 expression [94]. In BeWo cells, exposure to hypoxia increases GLUT1 expression [149]. Chronic hypoxia *in vivo* with high altitude pregnancies reduces expression of GLUT1 in BM but not in MVM [150]. 

## 6. Integrating Signals: Nutrient Supply,**** Placental Transport, and Fetal Growth

Fetal overgrowth and IUGR are associated with increased perinatal morbidity and mortality. They are also intimately linked with increased risk for development of metabolic disease later in life. Changes in placental nutrient transport capacity with altered fetal growth, and regulation of placental transport functions by maternal and fetal signaling molecules, have been extensively studied (in villous explants, cultured trophoblast cells, and animal models). Measurements of circulating factors in human pregnancies complicated by abnormal fetal growth are of particular interest because, together with the information on factors regulating placental nutrient transport reviewed here, such data allows identification of key factors regulating placental function *in vivo*. It is important to note that because the placenta produces many cytokines and hormones that could act in paracrine or autocrine fashion, the circulating levels may not be representative of concentrations in the intervillous space. We have proposed that alterations in maternal nutrient supply, adipokine, cytokine, and hormone levels such as those found in common pregnancy complications can lead to modification in placental transport function and subsequently have effects on fetal growth. Signals of reduced maternal nutrient availability (e.g., low insulin and leptin, high adiponectin) lead to an overall reduction in nutrient delivery to the fetus and slow fetal growth, while maternal signals of good nutritional status (such as high insulin and leptin, low adiponectin) stimulate nutrient delivery and accelerate fetal growth. Candidate regulatory factors and their effects on placental macronutrient transport functions are summarized in Table 2. However, nutrient transporters are specifically regulated and regulatory factors may have opposing effects on different transport systems, while rendering other transporters unaffected. Integration of multiple stimuli is critical for adjusting placental function to accommodate maternal supply and fetal demand.

Trophoblast cells must integrate numerous, possibly divergent, maternal and fetal stimuli and modify cellular function accordingly. Three examples of key placental signaling pathways involved with integrating multiple signals and regulating nutrient transport are (1) STAT3, involved in modulation of amino acid transport by cytokines, (2) PPAR*γ* affecting expression of FABPs and FATPs, and (3)*  *mTOR signaling pathway regulating placental amino acid transport (Figure 2). The importance of these pathways has been examined using *in vitro* experimental models. Additionally, altered activity and/or expression of placental mTOR and PPAR*γ* have been demonstrated in human pregnancies complicated by altered fetal growth [50, 51, 53, 151]. The origins of altered fetal growth are likely multifactorial, investigating single component effects will shed light on individual components as potential contributing factors. Yet, how such components orchestrate a unified response, modify placental nutrient transport, and regulate fetal growth remains to be established.

## 7. Concluding Remarks

Alterations in fetal development and growth have been associated with lifelong adverse health consequences. Since fetal growth and placental nutrient transport are closely linked, a cohesive knowledge of placental nutrient transport regulation will most certainly bring us closer to understanding those mechanisms underlying altered fetal growth. As reviewed in this paper, research has predominantly focused upon how individual factors, known to change in pregnancies complicated by pathological fetal growth, affect nutrient transport across the syncytiotrophoblast. This research resulted in the discovery of novel mechanisms involved in the regulation of placental nutrient transport. However, further work is needed which elucidates how multiple fetal, maternal, and placental signals are integrated and together regulate nutrient transport from mother to fetus. 

## Figures and Tables

**Table 1:** Changes in BM or MVM activity or placental expression of nutrient transporters and lipases in pregnancies complicated by abnormal fetal growth. Arrows indicate direction of change compared to normal fetal growth.

	Activity in BM	Activity in MVM	Expression	Refs.
IUGR				
System A	*↔*	↓		[35, 40]*
System L	↓	↓		[63]
Lysine	↓	*↔*		[63]
Taurine**	↓	↓	*↔*	[64, 65]
Glucose	*↔*	*↔*	*↔*	[35]
Lipoprotein lipase		↓	↑ or *↔*	[66–68]
Endothelial lipase			↓	[68]
LGA/macrosomia				
System A	*↔*	↑ or ↓	↑	[69–71]
System L	*↔*	↑ or *↔*		[69, 71]
Lysine	*↔*	*↔*		[69]
Taurine	*↔*	*↔*		[69]
Glucose	↑	*↔*	↑	[72]
Lipoprotein lipase		↑	*↔*	[66]

Increased (↑), decreased (↓), or unchanged (*↔*) transporter activity or expression in placenta from pregnancies with IUGR or fetal overgrowth as compared to normally grown fetuses. *[35] measured reduced system A activity in preterm IUGR but not term IUGR. **Effect only on sodium-independent uptake in BM and sodium-dependent uptake in MVM.

**Table 2:** Factors altered in pregnancy complications and their effect on placental nutrient transport.

Factor	Pregnancy complication	Amino acids	Fatty acids	Glucose	Refs.
CRH	**↑** IUGR (f) **↑** SGA (m)	**↓** System A		**↑** GLUT1 **↓** GLUT3	[61, 73–75]

Glucose	**↑** GDM (m)	**↑** System L **↓** Taurine		**↓** Uptake	[46, 76, 77]

IGF-I	**↓** IUGR (m) **↑** LGA (f)	**↑** System A	**↓** LPL activity		[21, 46, 78–80]

IL-6	**↑** Obesity (m)	**↑** System A	**↑** Lipid accumulation		[47, 81, 82]

Insulin	**↑** LGA (f)** **↑** GDM (m)	**↑** System A **↑** System L	**↑** LPL activity*	**↑** Uptake*	[21, 43–46, 79, 83–86]

Leptin	**↑** LGA (f) **↑** IUGR (m) **↑** Obesity (m) **↑** GDM (m)	**↑** System A	**↑** FAT		[21, 43, 45, 77, 82, 85, 87, 88]

Lipids	**↑** GDM (m) **↑** Obesity (m)	**↑** System A	**↓** LPL activity **↑** FABP4/5		[48, 77, 89–91]

mTOR	**↓** IUGR (p) **↑** Obesity (p)	**↑** System A **↑** System L **↑** Taurine			[46, 50, 53, 54]

Oxidative stress	**↑** IUGR	**↓** Taurine		**↓** Uptake	[65, 92–94]

Increased (↑), decreased (↓), and unchanged (*↔*) circulatory levels or placental activity/expression of nutrient transporters in pregnancies with IUGR or fetal overgrowth as compared to normally grown fetuses. Factors altered in maternal (m) circulation, fetal (f) circulation, or placenta (p). The effect of listed factors on the activity or expression of nutrient transporters has been determined *in vitro* in term placenta. SGA: small for gestational age. *Conflicting findings have been reported; see text for discussion. **Conflicting findings have been reported; for instance see [95].
